# The Clinical and Economic Value of Incorporating Monocyte Distribution Width Testing in the Detection of Occult Sepsis

**DOI:** 10.36469/001c.162383

**Published:** 2026-06-09

**Authors:** Lopamudra Das, Melissa Naiman, Jeff Radcliff, Mary Anne Sullivan Lofstrom, Shawn Schwartz, Matia Saeedian, William Ngantung, Sana Mirza

**Affiliations:** 1 Beckman Coulter, Inc., Brea, California; 2 BluePath Solutions, Los Angeles, California

**Keywords:** sepsis detection, sepsis screening, monocyte distribution width, emergency department triage, cost-consequence model, occult sepsis, health economics

## Abstract

**Background:**

Sepsis is a leading cause of morbidity, mortality, and increased healthcare costs. Early recognition is critical, yet occult sepsis often evades standard screening, delaying care and worsening outcomes. Monocyte distribution width (MDW), a novel blood biomarker automatically reported with a routine complete blood cell count (CBC), has emerged as a promising tool for early detection of occult sepsis in adult patients in the emergency department (ED).

**Objectives:**

To evaluate the clinical and economic impact of incorporating MDW into standard ED sepsis screening vs standard of care (SoC).

**Methods:**

This study utilized a cost-consequence model to evaluate the clinical and economic impact of incorporating MDW into standard ED sepsis screening compared with standard screening (SoC) alone. Adults presenting to the ED and meeting sepsis criteria within 24 hours of CBC collection were stratified by sepsis suspicion level (no, low, high) and MDW values (normal, elevated). Outcomes, including time-to-antibiotics, hospital and intensive care unit (ICU) length of stay (LOS), ICU admission, mortality, and readmissions, were estimated using trial data and regression models from published literature. Costs were assessed from a US provider perspective in 2024 US dollars.

**Results:**

In the base case, SoC + MDW testing was associated with lower costs per patient than SoC (36 340vs37 703; savings of $1363). Savings were driven by reduced ward and ICU LOS, particularly among patients with no or low suspicion of sepsis (eg, ICU LOS 0.30 vs 0.44 days; ward LOS 2.22 vs 2.75 days). Mortality was also reduced (0.03 vs 0.06 deaths per patient). While ED LOS increased in the MDW arm, inpatient savings offset these costs. Scenario analyses confirmed greater savings when unadjusted time-to-antibiotic data were applied. These findings are consistent with prior economic evaluations of early sepsis diagnostic tools and support the role of MDW in reducing downstream resource utilization, particularly among patients with occult presentations.

**Conclusions:**

MDW was found to be a low-cost tool that can strengthen sepsis screening by flagging cases earlier, especially in occult sepsis. Because it is reported with a routine CBC, MDW can be readily implemented and may improve outcomes while reducing costs.

## INTRODUCTION

Sepsis, defined as a life-threatening organ dysfunction caused by a dysregulated host response to infection, remains a leading cause of morbidity and mortality.^1^ It may progress to septic shock, a subtype of sepsis characterized by circulatory and cellular/metabolic dysfunction, with mortality rates of approximately 30% to 50% despite modern care.^2^ In 2021 alone, there were an estimated 166 million cases of sepsis, with over 21 million sepsis-related deaths, making up 31.5% of all global deaths.^3^ In the United States, sepsis contributes to roughly 1.7 million hospitalizations and 270 000 deaths annually.^4^

While sepsis is often associated with older adults, it can strike previously healthy people with little warning. Surprisingly, recent data show that previously healthy adults hospitalized with sepsis face nearly twice the risk of death than those with comorbidities, even after adjusting for illness severity.^5^ Sepsis also affects infants, children, and young adults. The World Health Organization estimates that nearly half of all sepsis cases globally occur in children under 5 years of age,^6^ with pediatric sepsis accounting for approximately 72 000 US hospitalizations in 2016.^7^ A nationwide study of young adults (20-44 years) in Spain reported an in-hospital mortality rate of 24% for sepsis.^8^ Survivors, particularly younger patients, often experience long-term physical, cognitive, and quality-of-life impairments, making early recognition critical for reducing both acute and long-term morbidity.

Early recognition and treatment of sepsis are critical for improving outcomes, with the 2021 Surviving Sepsis Campaign guidelines emphasizing that each hour of delayed antibiotic therapy increases mortality by 7% to 9%.^9,10^ Additionally, delays in antibiotic administration are linked to longer hospital stays and increased healthcare costs.^11,12^ These findings have informed national quality measures such as the Centers for Medicare & Medicaid Services (CMS) SEP-1 bundle, which standardizes early sepsis management, linking hospital reimbursement to adherence with evidence-based protocols. SEP-1 compliance is associated with better clinical outcomes and shorter hospital stays,^13^ though concerns about overtreatment and antibiotic stewardship remain.^14,15^ However, early detection of sepsis remains challenging due to its nonspecific symptoms. Patients may present to healthcare providers with nonspecific complaints such as fever, fatigue, or tachycardia, which may indicate sepsis but may also stem from common infections or other common illnesses.

Occult sepsis, where immune dysregulation and organ dysfunction are evolving but patients do not exhibit signs and symptoms consistent with standard sepsis screening criteria, poses a particular challenge, especially in younger patients, who often compensate physiologically, maintaining stable blood pressure and mental status even as sepsis progresses.^16^ This can mask the severity of illness, delaying recognition until rapid deterioration occurs. Studies estimate that 8% to 21% of sepsis cases are initially misdiagnosed, leading to higher rates of organ failure and death.^17^

Beyond its human toll, sepsis also imposes a high clinical and economic burden on healthcare systems. It is consistently ranked among the costliest conditions treated in hospitals,^12^ with an average cost per hospital-treated sepsis patient exceeding $30 000.^18^ Total US expenditures for sepsis care, including hospitalizations and post-acute services, reach tens of billions of dollars annually.^4^ Sepsis cases that are not promptly recognized at hospital presentation require more intensive care and incur higher costs. One study found that sepsis cases not present on admission, which included both missed recognition and new onset during hospital presentation, resulted in nearly threefold higher hospital costs compared with cases documented at admission (~$51 000 vs ~$18 000 per case, respectively).^12^

Traditional screening criteria (Systemic Inflammatory Response Syndrome [SIRS], quick Sequential Organ Failure Assessment [qSOFA], and other early warning scores) have imperfect sensitivity and can yield false negatives, especially in the initial stages of infection.^19^ Beyond these scoring systems, the diagnostic tools commonly used for sepsis detection face additional challenges. Blood cultures often require 24 to 72 hours for results. This delay can hinder timely intervention, especially given that approximately one-third of patients with suspected sepsis are blood culture negative, leading to potential misdiagnosis and inappropriate treatment.^20,21^ Biomarkers like procalcitonin and C-reactive protein are frequently used but have limitations. C-reactive protein lacks specificity, as its levels can be elevated in a variety of noninfectious inflammatory conditions.^22,23^ Procalcitonin, although more selective for bacterial infections, reaches peak diagnostic accuracy only around days 7 to 10 of illness, limiting its value in early detection.^22,23^ These tests often require longer turnaround times due to the complex nature of sample processing and analysis, which can significantly delay critical treatment initiation in patients with sepsis.^24,25^ In addition to clinical drawbacks, traditional diagnostic pathways carry considerable financial implications—blood culture panels and inflammatory marker tests are costly, and delays in diagnosis often lead to prolonged hospitalizations, higher intensive care unit (ICU) utilization, and increased overall healthcare spending.^4^ Given these limitations, there is a pressing need for diagnostic tools that can deliver rapid, reliable results without added cost or complexity.

To address these challenges, novel diagnostic approaches have emerged as promising tools in the rapid detection of sepsis. Monocyte distribution width (MDW) is a hematological biomarker that measures monocyte size variation, which increases during early immune response to infection.^26^ Unlike traditional sepsis screening methods, MDW is automatically reported as part of a routine complete blood cell count (CBC), providing real-time diagnostic insights without additional cost or workflow changes. While MDW does not assess sepsis severity, studies have shown that elevated MDW can predict sepsis development within the first 24 hours of presentation, even before overt symptoms appear.^24,27^ In a large multicenter trial, incorporating MDW into early emergency department (ED) evaluation increased sepsis detection odds by up to sixfold,^26^ and sensitivity as high as 87% has been reported using a cutoff around 20.1.^28,29^ Similarly, a 2022 prospective study further showed that MDW alone achieved an area under the curve of ~0.80 for diagnosing sepsis, and ~0.85 for septic shock, indicating strong diagnostic performance.^30^ By flagging potential sepsis cases earlier, MDW has the potential to accelerate intervention and reduce the clinical and economic burdens associated with delayed treatment.^31^

Despite the promise of many sepsis tools, economic analyses of sepsis interventions have shown mixed results, with cost-effectiveness varying based on treatment approach, patient populations, and hospital settings.^32^ Some interventions, such as early resuscitation bundles, have demonstrated cost savings, while others, like older molecular diagnostic tests, have increased costs with worsened outcomes.^32^ Many prior models have either focused singularly on economic outcomes or on clinical outcomes, without incorporating both.

This study estimates the clinical and economic impact of implementing MDW testing as part of the sepsis detection techniques employed in EDs. Using a cost-consequence modeling approach, we evaluate how MDW influences key clinical outcomes (eg, mortality, length of hospital/ICU stay) and healthcare costs when compared with standard of care (SoC) approaches to sepsis detection. By leveraging real-world data and economic modeling, this analysis provides a comprehensive assessment of MDW’s value in emergency care, including the extent to which it expediates detection and alleviates the burden of occult sepsis across both younger and adult populations.

## METHODS

### Model Overview

**Figure 1.**
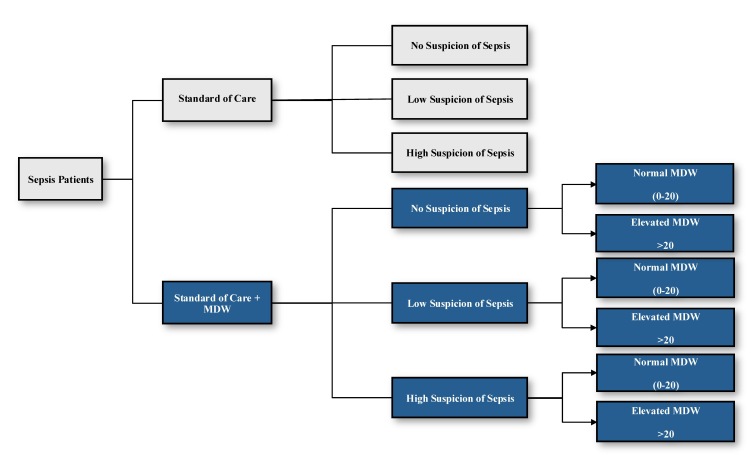
Decision-Tree Model Structure for Sepsis Triage with and without MDW Testing

This study utilized a decision-tree model to estimate the economic and clinical impact of SoC alone and in conjunction with MDW in triaging sepsis cases among a hypothetical cohort of adults with sepsis presenting to the ED (**Figure 1**). All patients were presumed to meet sepsis criteria within 24 hours of collecting a CBC, consistent with prior definitions,^33^ and were stratified based on sepsis suspicion level (no, low, high) and triage type (SoC, MDW). Patients assigned to MDW triage were further stratified based on the device’s accuracy in detecting sepsis (eg, whether the device returned a value of normal vs elevated MDW, which corresponded to missed vs detected cases in this study).

Within this framework, the impact of SoC ± MDW on time to antibiotic (TTA) administration and sepsis-related healthcare resource utilization were evaluated. Key outcomes included hospital and ICU rates of admission (and readmission), hospital and ICU length of stay (LOS), septic shock rates, and mortality rates. The analysis was conducted from the perspective of healthcare providers and modeled outcomes over a 1-month time horizon, which approximated the typical window of initial detection and treatment. As the goal of this study was to examine the utility of MDW in improving detection of occult sepsis, only results from patients assigned to the no/low suspicion groups were reported.

### Model Parameters

Modeled events (rates, durations) were obtained from a secondary analysis of data obtained from a large US academic hospital that recently adopted MDW testing in its ED.^34^ The dataset was first cleaned to adjudicate any misalignment between antibiotic administration, TTA documentation, and associated admissions/readmissions that could skew results. Cases with missing and/or ambiguous TTA documentation, and patients with LOS less than 48 hours were excluded to avoid bias toward unlikely or poorly documented sepsis cases. This final dataset provided a reliable foundation for the clinical inputs used in this model.

A full list of clinical inputs can be found in **Table S1**. The primary predictor of model outcomes was TTA, defined as the average time from ED triage to first antibiotic administration. Downstream effects included ICU/hospital admissions, associated LOS, septic shock rates, and mortality rates. Model inputs were primarily sourced from the clinical trial dataset referenced above^34^ or from regression-based estimates, except for hospital readmission rates, which were obtained from a prior observational study conducted among patients with sepsis.^12^ For normal MDW patients, who served as the baseline group, ICU rates, septic shock rates, mortality rates, hospitalization LOS, and ED LOS reported in the trial dataset were used directly in the analysis. For the elevated MDW patients, while ICU rates and ED LOS directly observed in the trial dataset were utilized, other outcomes such as hospital LOS, septic shock rates, and mortality rates used in the analysis were not. Instead, these outcomes were modeled using the change in TTA values observed between the elevated and normal MDW patient groups and regression coefficients derived from Ferrer et al,^35^ which evaluated the impact of TTA on sepsis outcomes among 165 ICUs in Europe, the United States, and South America. In Ferrer et al,^35^ time to antibiotic reported in this study ranged hourly from 1 hour to 6+ hours, and its influence on clinical outcomes such as hospital LOS, mortality, or septic shock rates were plotted to obtain these linear regression coefficients (see **Table S7** for further details). For example, from **Table S7**, a regression coefficient of 0.3214 was estimated for hospital LOS. The difference in mean TTA between patients with normal (8.92 hours) and elevated MDW (5.69 hours) patients in the clinical trial dataset was calculated (-3.23 hours). This difference was multiplied by the hospital LOS regression coefficient (-3.23 hours × 0.3214 =  -1.04 days) and added to the observed baseline hospital LOS (10.59 days), which was of the normal MDW patients, to estimate the hospital LOS for patients with elevated MDW (10.59 days -1.04 days = 9.56 days for elevated MDW patients). These adjustments allowed us to determine the net difference in TTA between treatment arms and to translate this to a net difference in outcomes among patients (see **Figure S1** for further details on how these equations were applied). ICU LOS was estimated as a proportion of total hospitalization LOS by applying TTA values reported from the trial dataset to ICU LOS and hospitalization LOS regression equations (**Table S7**). This proportion was then multiplied to the previously calculated hospital LOS to estimate ICU LOS. To ensure internal consistency, assumptions were made regarding the equivalence of normal MDW and SoC patients, use of suspicion level as a proxy for severity, and the application of readmission rates to all hospitalized patients.

Cost inputs for each clinical event were obtained from publicly available sources **(Table 1)**. Daily ICU and mechanical ventilation costs were obtained from published literature,^36^ while general hospitalization costs were sourced from the Kaiser Family Foundation.^37^ Readmission costs were estimated from a prior study examining the costs incurred by patients with sepsis.^12^ The cost of MDW implementation was provided by the manufacturer (data on file, 2024) and amortized over a 5-year time horizon to reflect equipment lifespan and allow annualized cost comparisons. All costs were estimated from the US provider perspective and inflation adjusted to 2024 US dollars using the medical care Consumer Price Index as reported by the Bureau of Labor Statistics (inflation factors are listed in **Table 1**) as needed before implementation.

**Table 1.** Cost Inputs for Sepsis-Related Clinical Events

**Resource**	**Published Value**	**Cost Year**	**Inflation Factor**	**Model Input Value**	**Source**
IP cost per day	$2883	2021	1.07	$3074	KFF^37^
ED cost per patient bed-hour	$58	2011	1.40	$81	Schreyer and Martin, 2017^38^
ICU cost per day	$4490	2002	1.96	$8805	Dasta et al, 2005^36^
MDW software cost	$8100	2024	1.00	$8100	Manufacturer, data on file, 2024
MDW machine cost	$60 076	2024	1.00	$60 075	Manufacturer, data on file, 2024
Incremental cost of septic shock	$13 660	2016	1.21	$16 499	Paoli et al, 2018^12^
Severe sepsis readmission cost	$15 717	2016	1.21	$18 984	Paoli et al, 2018^12^
Septic shock readmission cost	$18 587	2016	1.21	$22 450	Paoli et al, 2018^12^

Abbreviations: ED, emergency department; IP, inpatient; MDW, monocyte distribution width.

All data used in this study were obtained from de-identified and/or publicly accessible sources. The clinical inputs applied to the model from de-identified patient-level data were reviewed and approved by an external and University of Kansas Institutional Review Board (No. 21-STOC-10, Linking Novel Diagnostics with Data Driven Clinical Decision Support in the Emergency Department, September 23, 2023, ClinicalTrials.gov: NCT05335135); the full methodology and analysis of that dataset has been published previously.^34^ All other inputs were sourced from public and/or scientific literature. As a secondary analysis that did not collect any new data from human subjects, no ethical review or IRB approval was required.

### Supplemental Analyses

In addition to the base case described above, a deterministic one-way sensitivity analysis was performed to evaluate the uncertainty and robustness of our model results. Key inputs in the base case were varied by ±25% to assess their impact on clinical outcomes and identify key cost drivers within the current model design. A scenario analysis was also performed to explore whether using unadjusted inputs (rather than the Ferrer-derived approach^35^) influenced our study results. In this scenario analysis, the trial-reported TTA values were directly applied to generate estimates for hospital LOS, ICU, mortality, and septic shock (without any baseline adjustment, as in the base case analysis).

A multi-way sensitivity analysis was also performed to explore the variation in TTA and related outcomes that may be observed in different healthcare institutions. Key clinical inputs such as mortality and septic shock rates as well as hospitalization and ED LOS were varied by ±10% and ±25% from base-case values for both the SoC group and the MDW group.

Separately, a budget impact analysis was conducted to assess the financial implications of implementing MDW diagnostics in 100% of centers. This analysis assumed 1000 patients presented to the ED monthly, with a sepsis prevalence of 0.3%, consistent with estimates reported by the National Hospital Ambulatory Medical Care Survey.^39^

## RESULTS

The model results show that the implementation of MDW testing led to mixed clinical outcomes in the detection of occult sepsis (**Table 2**). Among patients with no suspicion of sepsis, implementing MDW led to 0.05 fewer hospitalizations per patient (17% reduction; 0.25 vs 0.30), reduced inpatient LOS by 0.53 days (19% reduction; 2.22 vs 2.75 days), and ICU LOS by 0.13 days (32% reduction; 0.30 vs 0.44 days) compared with SoC alone. Conversely, ED LOS increased by 1.29 hours (58% increase; 3.50 vs 2.21 hours). Similar trends were observed for patients with low suspicion of sepsis, where implementing MDW led to 0.01 fewer ICU admissions per patient, (6% reduction; 0.15 vs 0.16), reduced inpatient LOS by 0.28 days (6% reduction; 4.10 vs 4.38 days), and ICU LOS by 0.06 days (15% reduction; 0.34 vs 0.40 days). However, ED LOS was increased by 3.5 hours (73% increase; 8.30 vs 4.80 hours).

**Table 2.** Clinical Outcomes Associated with SoC ± MDW Among Adults with Occult Sepsis

	**No Suspicion**			**Low Suspicion**		
**SoC**	**MDW**	**Δ**	**SoC**	**MDW**	**Δ**	
ICU count	0.13	0.10	−0.03	0.16	0.15	−0.01
Septic shock count	0.04	0.04	−0.01	0.10	0.10	0.00
Mortality count	0.02	0.01	−0.01	0.02	0.01	−0.02
Readmission count	0.03	0.03	0.00	0.07	0.07	0.00
Hospitalization count	0.30	0.25	−0.05	0.59	0.58	−0.01
IP LOS, days	2.75	2.22	−0.53	4.38	4.10	−0.28
ED LOS, hours	2.21	3.50	1.30	4.80	8.30	3.49
ICU LOS (days)	0.44	0.30	−0.13	0.40	0.34	−0.06

Abbreviations: ED, emergency department; ICU, intensive care unit; IP, inpatient; LOS, length of stay; MDW, monocyte distribution width; SoC, standard of care.

Note: All reported outcomes were model-derived.

**Figure 2.**
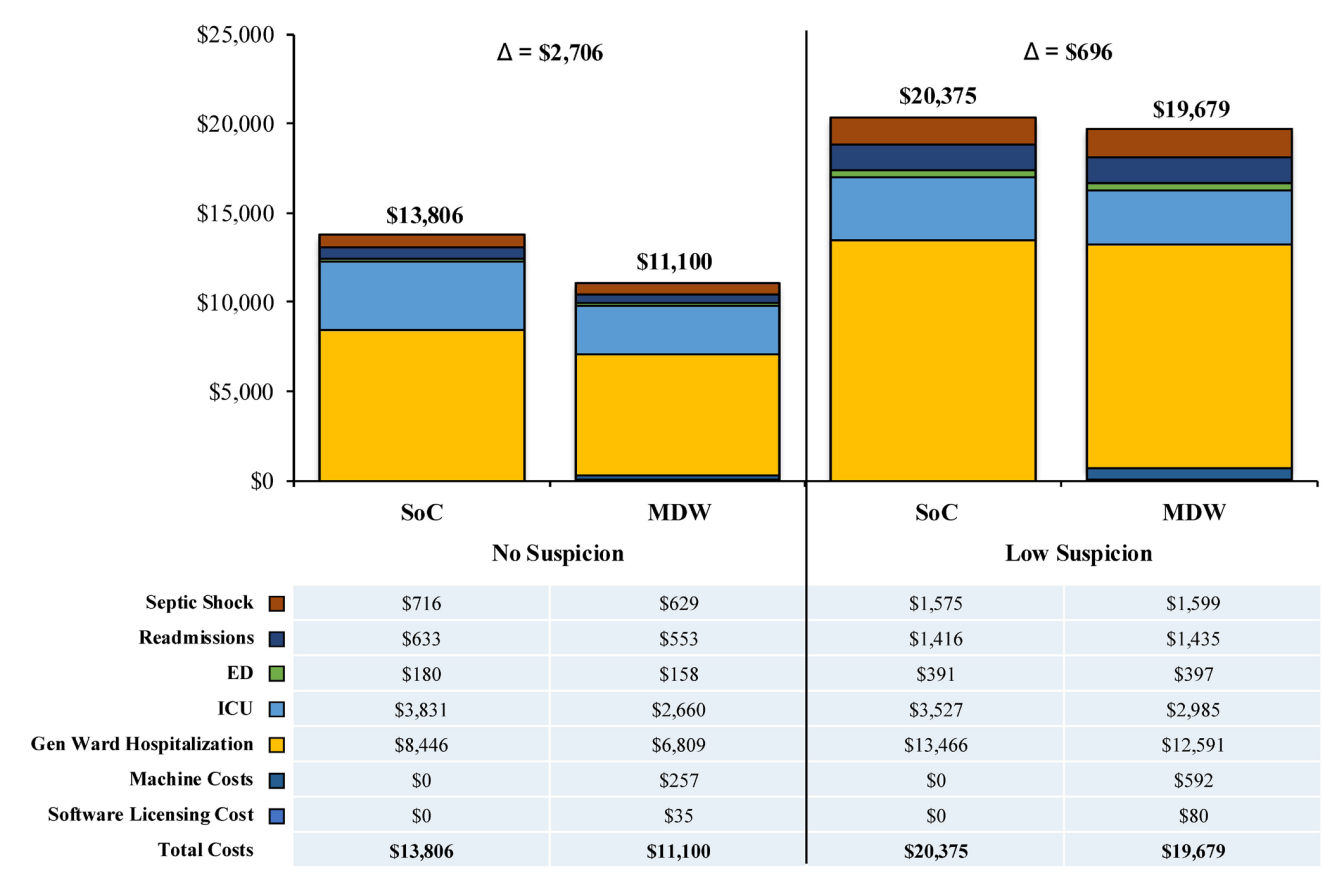
Costs for SoC Alone vs SoC + MDW Among Patients with Occult Sepsis (Adjusted TTA) Abbreviations: ED, emergency department; ICU, intensive care unit; MDW, monocyte distribution width; SoC, standard of care; TTA, time to antibiotic. Base case reflects clinical and economic outcomes using TTA values across arms. Stacked bar charts display per-patient costs stratified by cost category (general ward hospitalization, ICU, ED, readmissions, septic shock, machine costs, and software licensing costs) for patients with no suspicion (left panel) and low suspicion (right panel) of sepsis. Base case reflects clinical and economic outcomes using adjusted TTA values. Cost savings with MDW were $2706 per patient (no suspicion) and $696 per patient (low suspicion), driven primarily by reduced general ward hospitalization and ICU costs. ∆ = cost difference between SoC and MDW arms.

These patterns of (predominantly) lower healthcare resource utilization translated to a lower total cost per patient triaged with SoC + MDW vs SoC alone (**Figure 2**). MDW testing generated a cost reduction of $2706 per patient for those with no suspicion of sepsis (MDW vs SoC = $11 100 vs $13 806) and $696 per patient for those with low suspicion of sepsis (MDW vs SoC = $19 679 vs $20 375). The reduced costs of MDW were primarily driven by lower hospitalization costs (no suspicion = $6809 vs $8446; low suspicion = $12 591 vs $13 466) and ICU costs (no suspicion = $2660 vs $3831; low suspicion = $2985 vs $3527). Costs for septic shock and readmissions were also marginally lower in the MDW arms despite the added machine and licensing costs.

**Figure 3.**
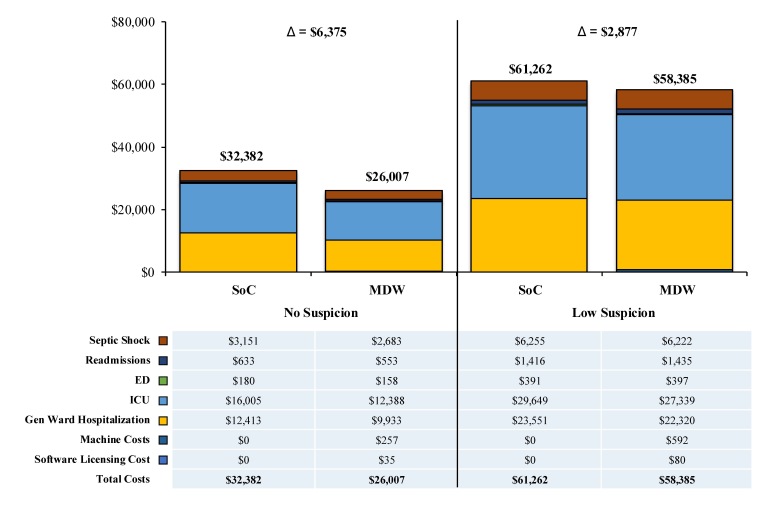
Costs for SoC Alone vs SoC + MDW Among Patients with Occult Sepsis (Unadjusted TTA) Abbreviations: ED, emergency department; ICU, intensive care unit; MDW, monocyte distribution width; SoC, standard of care; TTA, time to antibiotic. Scenario analysis using unadjusted TTA values. Stacked bar charts display per-patient costs stratified by cost category for patients with no suspicion (left panel) and low suspicion (right panel) of sepsis. Costs were systematically higher in each arm compared with the adjusted base case but yielded a similar pattern, with SoC + MDW less costly than SoC alone (Δ = $6375 per patient for no suspicion; Δ = $2877 per patient for low suspicion). Savings were predominantly driven by reduced hospitalization and ICU costs. Δ = cost difference between SoC and MDW arms.

Scenario analyses conducted using unadjusted clinical inputs (as an alternative to the base-case) produced systematically higher costs in each arm but yielded a similar pattern of results—wherein SoC + MDW was less costly than SoC alone (Δ = $2877-$6375 per patient), predominantly as a result of reduced hospitalizations and ICU stays (**Figure 3**). Sensitivity analyses indicated that hospital LOS (across various arms) was the most impactful driver of cost outcomes (**Figure S5**) with ICU admissions playing a smaller role. Budget impact analyses suggested that implementing MDW at scale would contribute cost savings, regardless of whether adjusted or unadjusted TTAs were applied (Δ = $1363-$4140 per patient, respectively) (**Figure S6; Table S6**).

## DISCUSSION

This study examined the economic and clinical impact of implementing MDW testing in ED sepsis screening. The primary objective was to determine whether adding MDW to standard sepsis evaluation can reduce healthcare costs per patient, while secondary objectives assessed improvements in key clinical outcomes such as TTA, LOS, ICU admissions, and readmissions. In the base-case analysis, MDW testing was associated with lower total costs per patient ($36 340 vs $37 703), driven largely by reduced general ward and ICU LOS, particularly among patients with low or no initial clinical suspicion for sepsis. The modeled cost savings were accompanied by reductions in ICU admissions, mortality, and septic shock, with no increase in readmissions. These findings suggest that MDW may support earlier recognition of sepsis and improve patient triage, particularly for patients with occult sepsis—an area of continued concern in sepsis management where delays in recognition contribute to increased mortality and cost.^16,17^

Among the most notable findings was the reduction in modeled inpatient and ICU LOS associated with MDW testing. For example, general ward LOS was 2.22 vs 2.75 days, and ICU LOS was 0.30 vs 0.44 days in the SoC + MDW vs SoC arms, respectively, for patients with low suspicion of sepsis. These reductions contributed meaningfully to per-patient cost savings and likely reflect earlier intervention for patients whose disease trajectory would otherwise escalate, thus aligning with well-established sepsis care principles: every hour of delay in effective antibiotic therapy has been associated with higher mortality risk in patients with septic shock,^9,10^ and clinical guidelines recommend treatment initiation within 1 hour of recognition.^40^ Although our model did not directly measure TTA, treatment timing was incorporated into the regression-based inputs that projected LOS and downstream outcomes. This link is supported by prior clinical data showing that MDW implementation can improve care delivery times. In a recent before-and-after cohort study, the introduction of MDW testing in an ED setting reduced median TTA from 3.7 hours to 2.2 hours,^41^ aligning with sepsis bundle targets such as CMS SEP-1.^13^ While the cohort study did not detect a benefit in mortality outcomes, the earlier intervention aligns with recognized best practices for time-sensitive management. Another notable finding of this analysis was the 73% increase in ED length of stay associated with the implementation of MDW testing. This has important downstream operational implications, particularly in high-volume EDs. Extending ED LOS in crowded EDs may strain hospital resources or impact care delivered to other patients within the department.

The observed reductions in healthcare costs with MDW are consistent with prior economic evaluations of early diagnostic tools for sepsis. A modeling study of a host gene expression test (HostDx Sepsis) projected a 31% per-patient cost reduction and improvement in hospital LOS and mortality, based on better triage and faster intervention.^42^ Similarly, a cost-effectiveness analysis of the IntelliSep Index reported that the tool out-performed procalcitonin-based approaches by improving diagnostic accuracy and reducing costs, largely by preventing escalation to critical care.^4^ Our MDW-based model shares these underlying themes; cost savings are concentrated in patients who would have otherwise deteriorated or required ICU level care but instead were identified and treated earlier. Notably, MDW offers practical advantages over other diagnostics due to its rapid availability and inclusion in a standard CBC with differential.^25,41^ A recent systematic review found that MDW has diagnostic performance comparable to procalcitonin, with the added benefit of faster turnaround and lower cost.^43^ Compared with previous MDW modeling efforts, the current analysis uses distinct modeling methodologies designed to enhance its realism and applicability. In the previous analysis, key parameters such as TTA were based on optimistic estimates, and clinical stratification was limited. Our current model instead uses real-world data to inform TTA distributions and stratifies patients by clinical suspicion level, providing greater insight into where MDW delivers value. These modifications allowed us to identify that the majority of savings and clinical improvements occur in patients with “occult” or low-suspicion sepsis. By modeling downstream outcomes based on these inputs, the analysis captures a more nuanced and clinically credible picture of MDW’s impact. It also demonstrates that even partial use of MDW can produce meaningful improvements in efficiency and outcomes, reinforcing its practicality for real-world use.

This study has several strengths. To our knowledge, it is the first health economic model of MDW informed by real-world clinical data. The model evaluates both economic and clinical outcomes and includes stratification by sepsis suspicion level, which aligns closely with how ED clinicians triage and manage patients. Additionally, MDW is uniquely positioned among sepsis diagnostics because it can be included in routine CBC testing, requires no additional workflow, and generates rapid results. These characteristics make it a low-barrier intervention with the potential for broad uptake. The model’s findings are also directionally consistent with prior studies of early diagnostics, suggesting that MDW may be a valuable tool for improving sepsis care without increasing cost or complexity.

### Limitations

Like all economic models, this analysis relies on assumptions to estimate the relationship between early detection and clinical outcomes. A key assumption in this model is that reductions in TTA are associated with improved clinical outcomes including reduced mortality, septic shock rates, and LOS. While the TTA inputs and clinical stratification were grounded in observed data, the link between earlier intervention and key clinical outcomes like mortality or LOS were derived from modeled relationships of TTA reduction rather than direct clinical trial evidence. These relationships are supported by prior literature but introduce inherent uncertainty. Additionally, the clinical inputs were sourced from a single ED center and may not generalize across settings with different practices or patient populations. As a result, the findings of this analysis do not capture variation across healthcare settings including differences in ICU admission thresholds or hospitalization practices. These differences will influence key model inputs and outcomes such as TTA or average hospital LOS, which ultimately affect total costs. To address this, a multi-way sensitivity analysis was conducted by varying key clinical inputs to reflect potential differences across healthcare settings. Results showed that throughout the different combination of scenarios, using MDW still resulted in cost-savings for occult sepsis patients in most scenarios, except when SoC inputs were decreased while MDW inputs were increased (see **Table S8** and **Table S9**). The model also focuses on short-term, hospital-based outcomes; it does not capture longer-term patient trajectories, quality-of-life impacts, or costs beyond a set number of days, despite the known long-term burden of sepsis, especially in younger survivors.^7,8^ Furthermore, while the analysis included equipment and software costs associated with MDW diagnostics, it did not consider implementation-related costs, such as training providers or adjusting workflows. Future analyses incorporating these costs may better capture the real-world economic impact of MDW implementation. These indirect one-time costs were not included in this analysis but should be considered by an institution when implementing new technologies. Lastly, variability in clinician awareness, interpretation, and response to MDW may influence real-world effectiveness.

Despite these limitations, the findings have important implications for ED sepsis management. MDW offers a pragmatic solution to a persistent clinical challenge; recognizing sepsis early, especially in patients with occult sepsis. By helping flag these cases earlier, MDW can support faster treatment and reduce escalation to critical illness, ultimately lowering costs and improving care efficiency. Hospitals seeking to improve performance on sepsis compliance or reduce avoidable ICU utilization may find MDW a valuable addition to their diagnostic capabilities. Additionally, MDW may support antimicrobial stewardship efforts by helping clinicians identify which patients are unlikely to benefit from empiric broad-spectrum antibiotics.

Future research should focus on prospective studies evaluating MDW’s impact on clinical decision-making and patient outcomes. While this model demonstrates potential efficiency and cost gains, real-world effectiveness depends on how MDW influences behavior. This includes whether clinicians act on the results to initiate treatment earlier or tailor diagnostic workups accordingly. Studies examining how MDW affects clinical workflow, antibiotic prescribing patterns, and patient throughput in the ED would help clarify its value further. Research into how MDW performs when integrated with other diagnostic tools may also provide insight into optimal sepsis screening strategies. Additionally, future analyses should investigate the cost-effectiveness of MDW from a health system or societal perspective. These approaches would help determine the extent to which the clinical and economic benefits of MDW are realized for patients or providers and determine the long-term value of MDW. Specifically, cost-effectiveness analyses could evaluate the impact of MDW on long-term benefits such as survival or quality of life and determine its economic value. Finally, long-term studies are needed to evaluate whether earlier detection via MDW reduces downstream morbidity, readmissions beyond 30 days, or post-sepsis syndrome.

## CONCLUSIONS

This study provides new real-world evidence supporting the clinical and economic value of MDW testing for early sepsis identification in the ED. By enabling more timely intervention, especially for patients with occult sepsis, MDW may help reduce inpatient resource use, lower costs, and improve patient outcomes. Its seamless integration into routine labs and rapid turnaround make it a feasible tool for widespread adoption. While additional research is needed to confirm patient-level outcome benefits, these findings suggest that MDW is a practical innovation that can strengthen early sepsis recognition and help close gaps in emergency care delivery.

### Declarations

L.D., M.N., J.R., S.S., and M.S.L. are employees of Beckman Coulter and hold equity interests in the company. M.S., W.N., and S.M. are employees of BluePath Solutions, which was contracted by Beckman Coulter to support the model design, execution, and analysis, and to assist with manuscript preparation.

## Supplementary Material

Online Supplementary Material
