# Supplementary material for: The Clinical and Economic Value of Incorporating Monocyte Distribution Width Testing in the Detection of Occult Sepsis
Source: J Health Econ Outcomes Res. 2026 Jun 9;13(1):218–25. doi: 10.36469/001c.162383 (PMC13259569; doi:10.36469/001c.162383)
Supplement: Online Supplementary Material [file jheor_2026_13_1_162383_348533.pdf]

Journal of Health Economics and Outcomes Research

Online Supplementary Material

The Clinical and Economic Value of Incorporating Monocyte Distribution Width Testing in the Detection of Occult Sepsis. *JHEOR*. 2026;13(1):218-225. doi:10.36469/jheor.2026.162383

Table S1: Clinical Inputs 1

Table S2: Clinical Outcomes Among All Sepsis Levels (Unadjusted TTA) 3

Table S3: Clinical Outcomes Among Patients with No Suspicion of Sepsis (Unadjusted TTA) 3

Table S4: Clinical Outcomes Among Patients with Low Suspicion of Sepsis (Unadjusted TTA) 4

Table S5: Clinical Outcomes Among All Sepsis Levels (Adjusted TTA) 4

Table S6: Budget Impact Analysis – Cost Comparison Breakdown (Adjusted TTA) 5

Table S7: Inputs from Ferrer et al[34] to Calculate Regression Coefficients 5

Table S8: Multi-Way Sensitivity Analysis – No Suspicion of Sepsis 6

Table S9: Multi-Way Sensitivity Analysis – Low Suspicion of Sepsis 6

Figure S1: Equations Used to Estimate Clinical Outcomes From Time to Antibiotic Administration 7

Figure S2: Total Costs Among All Sepsis Levels 8

Figure S3: Total Costs Among Patients with No Suspicion of Sepsis 9

Figure S4: Total Costs Among Patients with Low Suspicion of Sepsis 10

Figure S5: One-Way Sensitivity Analysis 11

Figure S6: Budget Impact of MDW Implementation Across Uptake Levels: Base Case vs Scenario Estimates 12

This supplementary material has been provided by the authors to give readers additional information about their work.

This is an open-access article distributed under the terms of the Creative Commons Attribution 4.0 International License (CCBY-4.0). View this license's legal deed at http://creativecommons.org/licenses/by/4.0 and legal code at http://creativecommons.org/licenses/by/4.0/legalcode for more information.

Table S1. Clinical Inputs

**Average TTA**	**TTA, Hours**
Normal MDW/SoC	
No suspicion	8.92
Low suspicion	7.37
High suspicion	3.64
Elevated MDW	
No suspicion	5.69
Low suspicion	5.10
High suspicion	3.38
**ICU Rates*,[a]**	**ICU, %**
Normal MDW/SoC	
No suspicion	43.57
Low suspicion	26.99
High suspicion	35.56
Elevated MDW	
No suspicion	36.62
Low suspicion	24.33
High suspicion	29.22
**Hospitalization Rates***	**Hospitalization, %**
Normal MDW/SoC	
No suspicion	98.93
Low suspicion	98.37
High suspicion	100.00
Elevated MDW	
No suspicion	99.26
Low suspicion	98.80
High suspicion	99.75
**Mortality [a]**	**Mortality Rates**
Normal MDW/SoC	
No suspicion	7.50
Low suspicion	3.62
High suspicion	11.11
Elevated MDW	
No suspicion	2.49
Low suspicion	0.10
High suspicion	10.71

Septic Shock[a]	Septic Shock Rate, %	
Normal MDW/SoC		
No suspicion	14.29	
Low suspicion	15.94	
High suspicion	26.67	
Elevated MDW		
No suspicion	15.19	
Low suspicion	16.58	
High suspicion	26.74	
**Readmissions[b]**	**Readmission Rate, %**	
Low suspicion of sepsis	11.10	
Moderate/high suspicion	12.30	
Septic shock	13.60	
**Average Length of Stay [a]**	**Hospital LOS, Days**	**ED LOS, Hours**
Normal MDW/SoC		
No suspicion	10.59	7.26
Low suspicion	8.12	8.03
High suspicion	7.85	7.85
Elevated MDW		
No suspicion	9.56	7.71
Low suspicion	7.39	8.35
High suspicion	7.77	7.92

[*] Sourced using proprietary manufacturer internal trial dataset

[a] The model allowed users to toggle between trial data and estimated rates based on time to antibiotic administration (TTA) regression from Ferrer et al. (2014) [34]. See Table S3-S5 and Figure S2-S4 for results.

[b] Trial data did not report any readmissions, so the model used Paoli et al., 2018 to assign readmission rates [12]. Patients without organ dysfunction were assigned to the no suspicion group, those with severe sepsis to low or high suspicion groups, and patients with septic shock used the corresponding readmission probability.

Abbreviations: ED, emergency department; ICU, intensive care unit; LOS, length of stay; MDW, monocyte distribution width; SoC, standard of care.; TTA, time to antibiotic administration.

Table S2. Clinical Outcomes Among All Sepsis Levels (Unadjusted TTA)

	SoC	Normal MDW	Elevated MDW	MDW Total	Difference
ICU count	0.00	0.00	0.00	0.00	0.00
Septic shock count	0.63	0.18	0.46	0.64	0.01
Mortality count	0.38	0.11	0.24	0.35	-0.03
Readmission count	0.12	0.03	0.09	0.12	0.00
Hospitalization	0.00	0.00	0.00	0.00	0.00
IP LOS, days	12.83	3.71	8.55	12.25	-0.58
ED LOS, hours	7.78	7.78	5.77	13.55	5.77
ICU LOS, days	5.65	1.63	3.60	5.23	-0.42

Abbreviations: ED, emergency department; ICU, intensive care unit; LOS, length of stay; MDW, monocyte distribution width; SoC, standard of care.; TTA, time to antibiotic administration.

Table S3. Clinical Outcomes Among Patients with No Suspicion of Sepsis (Unadjusted TTA)

	SoC	Normal MDW	Elevated MDW	MDW Total	Difference
ICU count	0.00	0.00	0.00	0.00	0.00
Septic shock count	0.19	0.06	0.11	0.16	-0.03
Mortality count	0.12	0.04	0.06	0.09	-0.03
Readmission count	0.03	0.01	0.02	0.03	0.00
Hospitalization	0.00	0.00	0.00	0.00	0.00
IP LOS, days	4.04	1.17	2.07	3.23	-0.81
ED LOS, hours	2.21	2.21	1.30	3.50	1.30
ICU LOS, days	1.82	0.52	0.88	1.41	-0.41

Abbreviations: ED, emergency department; ICU, intensive care unit; LOS, length of stay; MDW, monocyte distribution width; SoC, standard of care.; TTA, time to antibiotic administration.

Table S4. Clinical Outcomes Among Patients with Low Suspicion of Sepsis (Unadjusted TTA)

	SoC	Normal MDW	Elevated MDW	MDW Total	Difference
ICU count	0.00	0.00	0.00	0.00	0.00
Septic shock count	0.38	0.11	0.27	0.38	0.00
Mortality count	0.23	0.07	0.14	0.21	-0.02
Readmission count	0.07	0.02	0.05	0.07	0.00
Hospitalization	0.00	0.00	0.00	0.00	0.00
IP LOS, days	7.66	2.21	5.05	7.26	-0.40
ED LOS, hours	4.80	4.80	3.49	8.30	3.49
ICU LOS, days	3.37	0.97	2.13	3.11	-0.26

Abbreviations: ED, emergency department; ICU, intensive care unit; LOS, length of stay; MDW, monocyte distribution width; SoC, standard of care.; TTA, time to antibiotic administration.

Table S5. Clinical Outcomes Among All Sepsis Levels (Adjusted TTA)

	SoC	MDW			Difference
		Normal	Elevated	Total	
ICU count	0.33	0.09	0.20	0.29	-0.03
Septic shock count	0.16	0.05	0.13	0.18	0.01
Mortality count	0.06	0.02	0.02	0.03	-0.02
Readmission count	0.12	0.03	0.09	0.12	0.00
Hospitalization	0.99	0.28	0.70	0.99	0.00
IP LOS, days	7.82	2.26	5.13	7.39	-0.42
ED LOS, hours	7.78	7.78	5.77	13.55	5.77
ICU LOS, days	0.92	0.26	0.48	0.75	-0.17

Abbreviations: ED, emergency department; ICU, intensive care unit; LOS, length of stay; MDW, monocyte distribution width; SoC, standard of care.; TTA, time to antibiotic administration.

Table S6. Budget Impact Analysis – Cost Comparison Breakdown (Adjusted TTA)

	Reference Scenario (0% MDW Utilization)			New Scenario (100% MDW Utilization)		
	**SoC**	**MDW**	**Total**	**SoC**	**MDW**	**Total**
Software licensing cost	$0	$0	$0	$0	$135	$1001
Machine costs	$0	$0	$0	$0	$135	$1001
Hospitalization	$24,024	$0	$24,024	$0	$22,718	$22,718
ICU	$8,056	$0	$8,056	$0	$6,566	$6,566
ED	$633	$0	$633	$0	$653	$653
Readmissions	$2,270	$0	$2,270	$0	$2,366	$2,366
Septic shock	$2,720	$0	$2,720	$0	$2,901	$2,901
**Total**	**$37,703**	**$0**	**$37,703**	**$0**	**$36,340**	**$36,340**

Abbreviations: ED, emergency department; ICU, intensive care unit; LOS, length of stay; MDW, monocyte distribution width; SoC, standard of care.; TTA, time to antibiotic administration.

Note: The *Reference Scenario* assumes 0% MDW uptake (standard of care only), while the *New Scenario* assumes 100% MDW uptake among patients presenting to the ED. The analysis models a population of 1,000 ED visits per month with a sepsis prevalence of 0.3%.

Table S7. Inputs from Ferrer et al[34] to Calculate Regression Coefficients

	Time to Antibiotic, Hours						
	**1.0**	**2.0**	**3.0**	**4.0**	**5.0**	**6.0**	**7.0**
N	4,728	4,595	3,020	1,734	1,037	640	2,239
Hospital mortality (%)	32.0	28.1	28.6	29.8	32.5	36.6	39.6
Septic shock (%)	69.6	62.7	61.2	60.4	66.0	68.9	61.3
Hospital LOS, days	13	10	10	11	12	12	14
ICU LOS, days	5.1	4.1	4.2	4.3	4.9	4.6	6.7

Abbreviations: ICU, intensive care unit; LOS, length of stay.

These inputs were plotted against time-to-antibiotic and a linear regression was performed to estimate regression coefficients for hospital mortality, septic shock, hospital LOS, and ICU LOS. The resulting coefficients were applied to time-to-antibiotic differences observed in the trial dataset to estimate modeled outcomes for patients with elevated MDW values. The resulting equations were as follows: Hospital LOS: 0.3214*(TTA) + 10.429; ICU LOS: 0.2321*(TTA) + 3.9143; Mortality Rate: 0.0155*(TTA) + 0.2628; Septic Shock Rate: -0.0028*(TTA) + 0.6539

Table S8. Multi-Way Sensitivity Analysis – No Suspicion of Sepsis

	MDW -25%	MDW -10%	Base Case	MDW +10%	MDW +25%
SoC +25%	-$6,665	-	-	-	-$3,431
SoC +10%	-	-$4,290	-	-$2,997	-
Base Case	-	-	-$2,707	-	-
SoC -10%	-	-$2,417	-	-$1,124	-
SoC -25%	-$1,982	-	-	-	$1,251

Abbreviations: MDW, monocyte distribution width; SoC, standard of care.

Note: Cells highlighted in green denote savings while using MDW diagnostics while cells in red denote costs while using MDW diagnostics.

Table S9. Multi-Way Sensitivity Analysis – Low Suspicion of Sepsis

	MDW -25%	MDW -10%	Base Case	MDW +10%	MDW +25%
SoC +25%	-$7,107	-	-	-	-$1,034
SoC +10%	-	-$3,261	-	-$832	-
Base Case	-	-	-$697	-	-
SoC -10%	-	-$561	-	$1,868	-
SoC -25%	-$359	-	-	-	$5,714

Abbreviations: MDW, monocyte distribution width; SoC, standard of care.

Note: Cells highlighted in green denote savings while using MDW diagnostics while cells in red denote costs while using MDW diagnostics.

Figure S1. Equations Used to Estimate Clinical Outcomes From Time to Antibiotic Administration (TTA)

Net difference in TTA between normal MDW and elevated MDW is calculated for each of the suspicion levels.

$$\boldsymbol{Normal\ MDW\ TTA - Elevated\ MDW\ TTA = Net\ Difference\ in\ TTA}$$

↓

The net difference in TTA is then multiplied by the **coefficients** from the published equations to calculate a net difference in outcomes between normal MDW and the elevated MDW for all three suspicion levels. Outcomes include septic shock rates, mortality rates, and hospitalization LOS.

$$\boldsymbol{Net\ Difference\ in\ TTA\ x\ Coefficient\ for\ Hospitalization, Mortality, or\ Septic\ Shock\ Equations = Net\ Difference\ in\ Rate\ or\ LOS}$$

↓

This calculated net difference in rate or LOS is then added to the normal MDW's corresponding rate or LOS to calculate a new adjusted rate for the elevated MDW groups across the three suspicion levels.

$$\boldsymbol{Net\ Difference\ in\ Mortality, Septic\ Shock\ Rates, or\ Hospital\ LOS + Normal\ MDW\ Rate\ of\ LOS = New\ Adjusted\ Rate\ or\ LOS}$$

Note: Published Equations and the **Coefficients** are as follows: Hospital LOS = 0.3214*(TTA) + 10.429; Mortality Rate = 0.0155*(TTA) + 0.2628; Septic Shock Rate = -0.0028*(TTA) + 0.6539
MDW, Monocyte Distribution Width; TTA, Time-to-Antibiotic; LOS, Length of Stay

Figure S2. Total Costs Among All Sepsis Levels

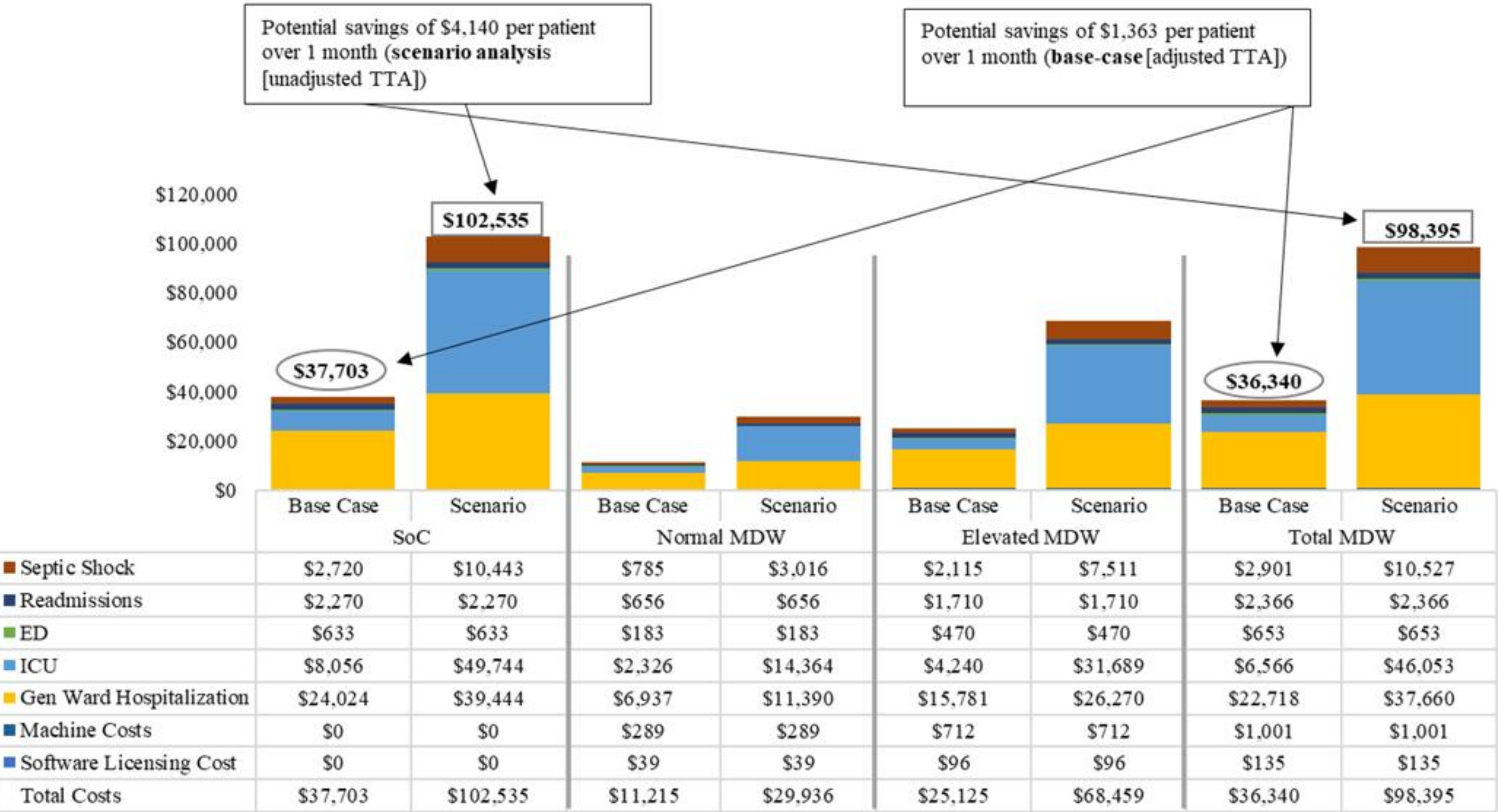

	SoC		Normal MDW		Elevated MDW		Total MDW	
	Base Case	Scenario	Base Case	Scenario	Base Case	Scenario	Base Case	Scenario
Septic Shock	$2,720	$10,443	$785	$3,016	$2,115	$7,511	$2,901	$10,527
Readmissions	$2,270	$2,270	$656	$656	$1,710	$1,710	$2,366	$2,366
ED	$633	$633	$183	$183	$470	$470	$653	$653
ICU	$8,056	$49,744	$2,326	$14,364	$4,240	$31,689	$6,566	$46,053
Gen Ward Hospitalization	$24,024	$39,444	$6,937	$11,390	$15,781	$26,270	$22,718	$37,660
Machine Costs	$0	$0	$289	$289	$712	$712	$1,001	$1,001
Software Licensing Cost	$0	$0	$39	$39	$96	$96	$135	$135
Total Costs	$37,703	$102,535	$11,215	$29,936	$25,125	$68,459	$36,340	$98,395

Note: *Base case* reflects clinical and economic outcomes using **adjusted TTA values** across arms. *Scenario* reflects outcomes using **unadjusted TTA values**.

Figure S3. Total Costs Among Patients with No Suspicion of Sepsis

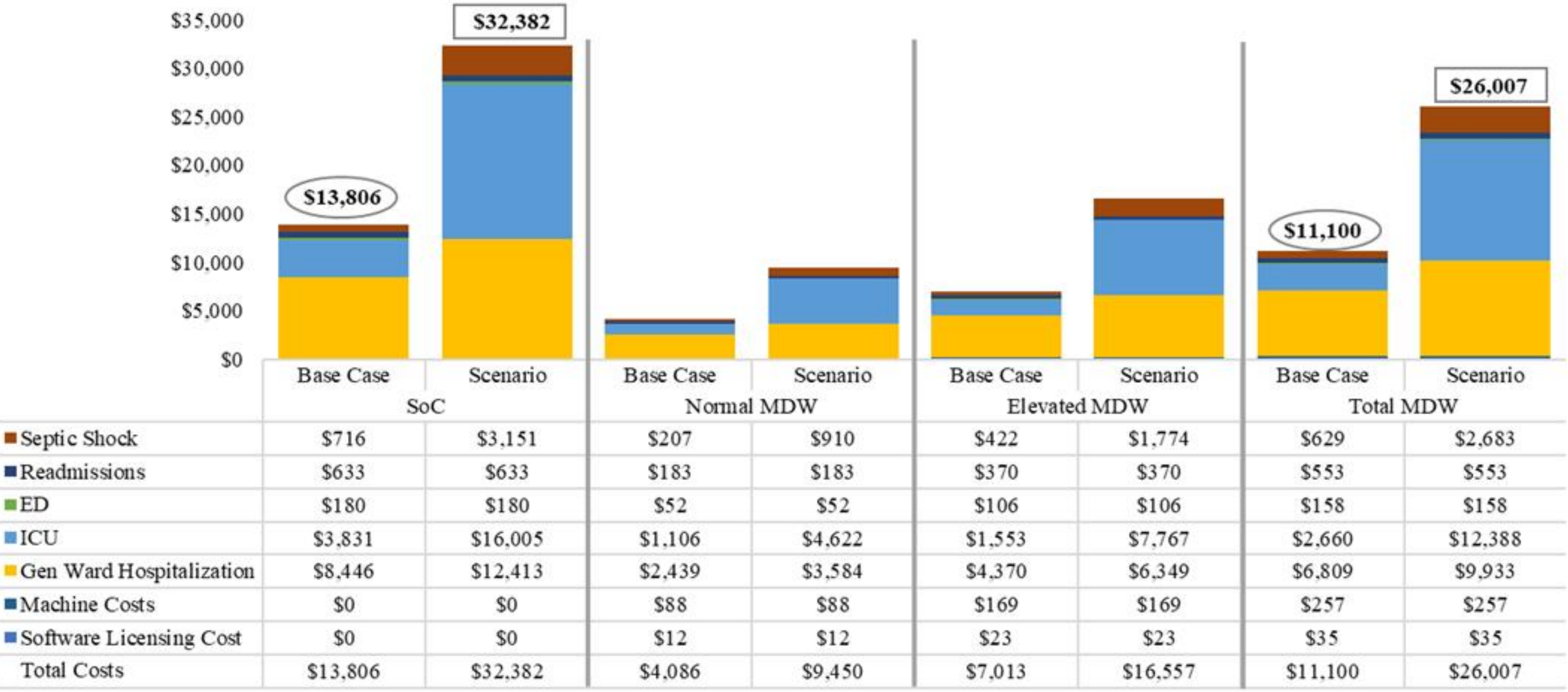

	SoC		Normal MDW		Elevated MDW		Total MDW	
	Base Case	Scenario	Base Case	Scenario	Base Case	Scenario	Base Case	Scenario
Septic Shock	$716	$3,151	$207	$910	$422	$1,774	$629	$2,683
Readmissions	$633	$633	$183	$183	$370	$370	$553	$553
ED	$180	$180	$52	$52	$106	$106	$158	$158
ICU	$3,831	$16,005	$1,106	$4,622	$1,553	$7,767	$2,660	$12,388
Gen Ward Hospitalization	$8,446	$12,413	$2,439	$3,584	$4,370	$6,349	$6,809	$9,933
Machine Costs	$0	$0	$88	$88	$169	$169	$257	$257
Software Licensing Cost	$0	$0	$12	$12	$23	$23	$35	$35
Total Costs	$13,806	$32,382	$4,086	$9,450	$7,013	$16,557	$11,100	$26,007

Note: *Base case* reflects clinical and economic outcomes using **adjusted TTA values** across arms. *Scenario* reflects outcomes using **unadjusted TTA values**.

Figure S4. Total Costs Among Patients with Low Suspicion of Sepsis

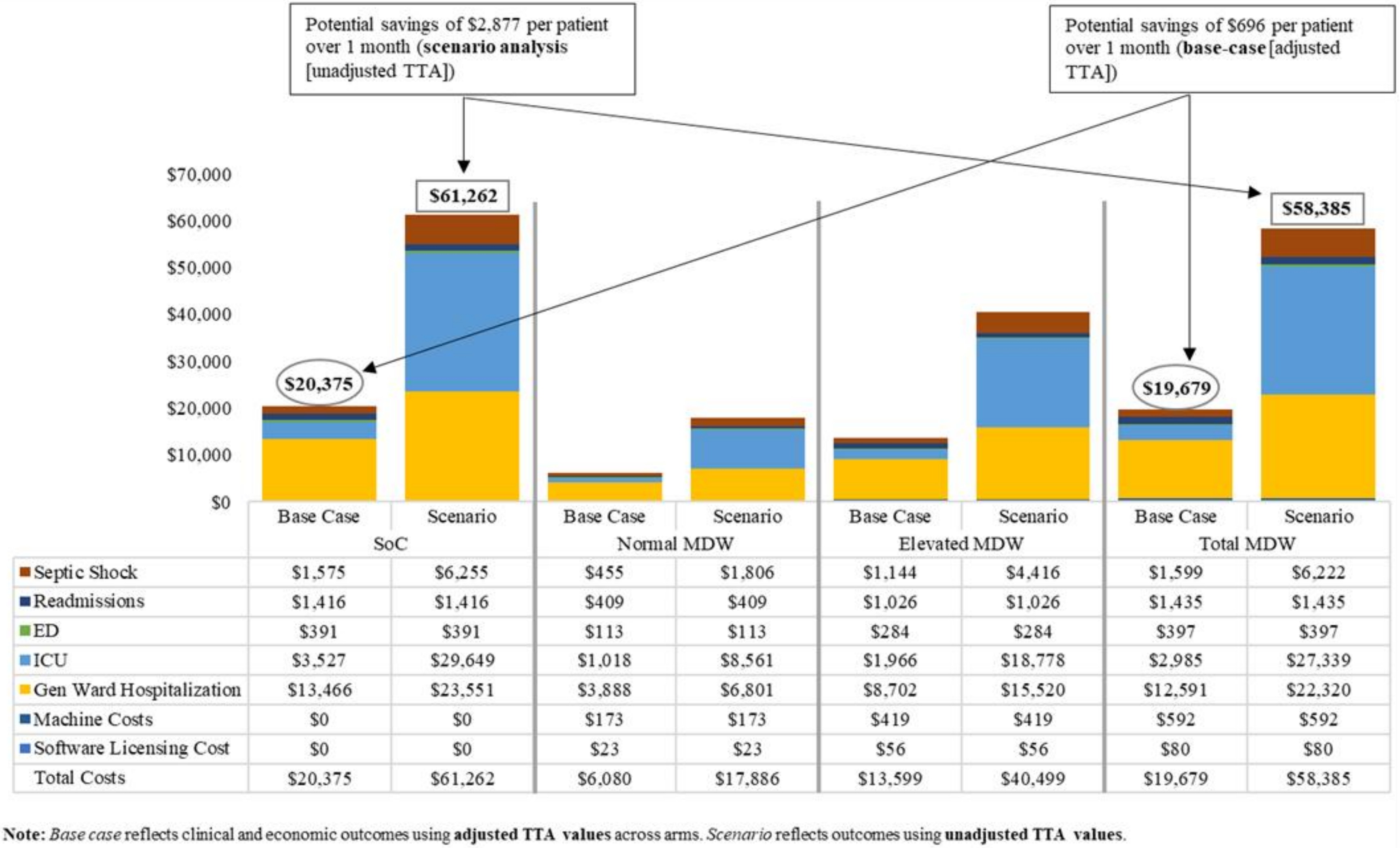

	SoC		Normal MDW		Elevated MDW		Total MDW	
	Base Case	Scenario	Base Case	Scenario	Base Case	Scenario	Base Case	Scenario
Septic Shock	$1,575	$6,255	$455	$1,806	$1,144	$4,416	$1,599	$6,222
Readmissions	$1,416	$1,416	$409	$409	$1,026	$1,026	$1,435	$1,435
ED	$391	$391	$113	$113	$284	$284	$397	$397
ICU	$3,527	$29,649	$1,018	$8,561	$1,966	$18,778	$2,985	$27,339
Gen Ward Hospitalization	$13,466	$23,551	$3,888	$6,801	$8,702	$15,520	$12,591	$22,320
Machine Costs	$0	$0	$173	$173	$419	$419	$592	$592
Software Licensing Cost	$0	$0	$23	$23	$56	$56	$80	$80
Total Costs	$20,375	$61,262	$6,080	$17,886	$13,599	$40,499	$19,679	$58,385

Note: *Base case* reflects clinical and economic outcomes using **adjusted TTA values** across arms. *Scenario* reflects outcomes using **unadjusted TTA values**.

Figure S5. One-Way Sensitivity Analysis

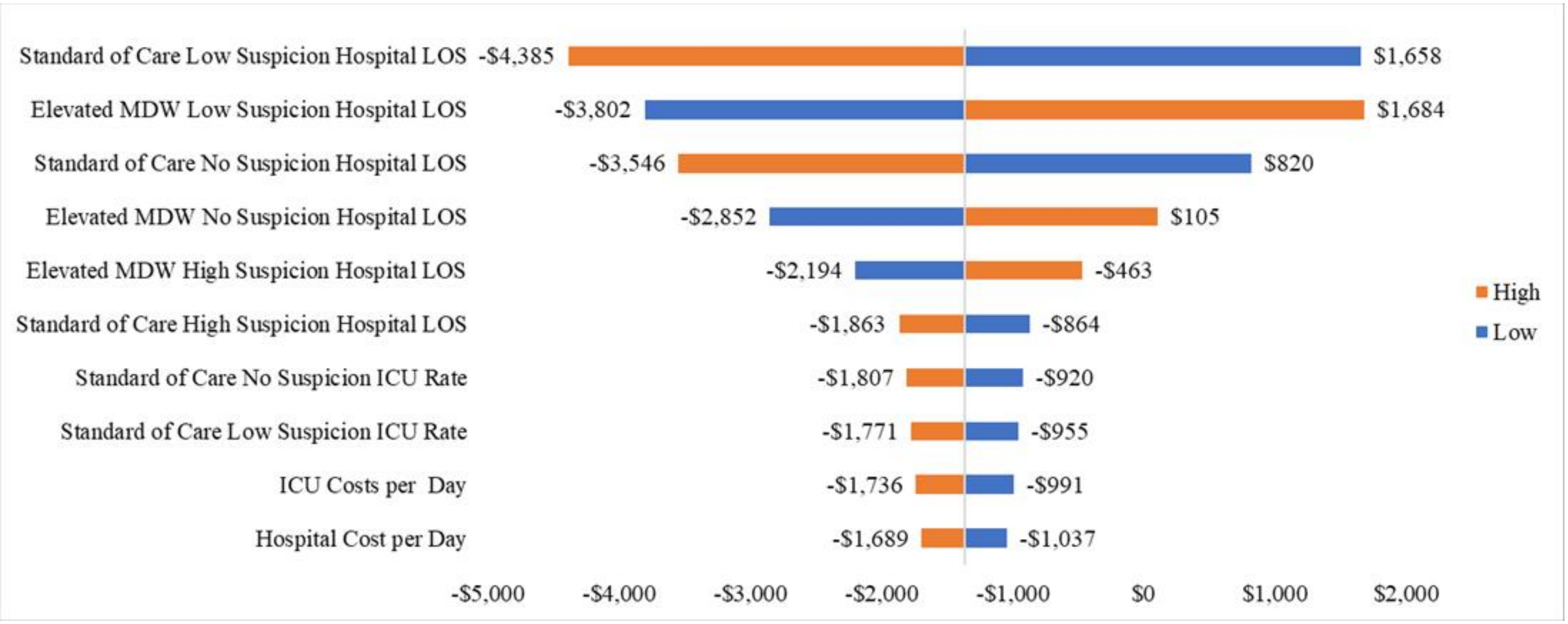

Note: Analyses were run by varying the model base case inputs by ±25% to determine the sensitivity of model inputs on the results. Values shown in this graph are the differences in total costs when inputs are varied by ±25%

Figure S6. Budget Impact of MDW Implementation Across Uptake Levels: Base Case vs Scenario Estimates

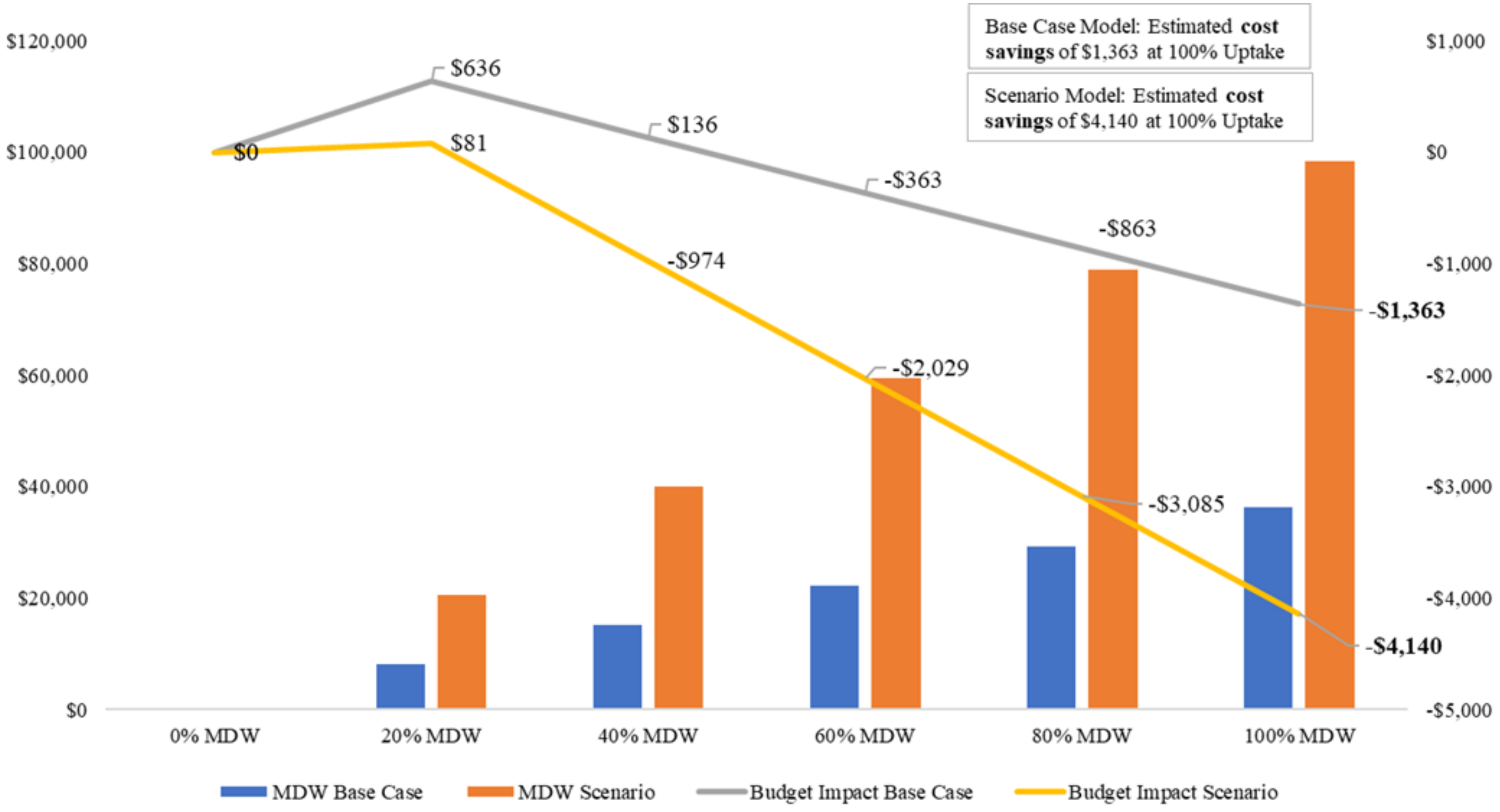

*Assumes there are 1,000 patients that enter the ED in a month, a sepsis prevalence of 0.3%
Note: *Base case* reflects clinical and economic outcomes using **adjusted TTA values** across arms. *Scenario* reflects outcomes using **unadjusted TTA values**
MDW, Monocyte Distribution Width; SoC, Standard of Care
